# First record of the genus *Plynnon* Deeleman-Reinhold, 2001 from China, with the description of a new species (Araneae, Phrurolithidae)

**DOI:** 10.3897/BDJ.10.e85029

**Published:** 2022-05-11

**Authors:** Chi Jin, Xiaoling Li, Feng Zhang

**Affiliations:** 1 School of Landscape and Ecological Engineering, Hebei University of Engineering, Handan, China School of Landscape and Ecological Engineering, Hebei University of Engineering Handan China; 2 The Key Laboratory of Zoological Systematics and Application, College of Life Sciences, Hebei University, Baoding, China The Key Laboratory of Zoological Systematics and Application, College of Life Sciences, Hebei University Baoding China

**Keywords:** Dionycha, diagnosis, taxonomy, Southeast Asia, Yunnan Province

## Abstract

**Background:**

*Plynnon* Deeleman-Reinhold, 2001 is a small phrurolithid spider genus distributed in Southeast Asia, with three currently known species: *P.longitarse* Deeleman-Reinhold, 2001 and *P.zborowskii* Deeleman-Reinhold, 2001 from Borneo and *P.jaegeri* Deeleman-Reinhold, 2001 from Sumatra.

**New information:**

*Plynnonaduncum* sp. n. (♂, ♀) from Yunnan Province, China is described, representing the northernmost record for the genus. Illustrations and morphological descriptions are provided.

## Introduction

The spider family Phrurolithidae Banks, 1892 includes 304 extant and three fossil species in 20 genera (Dunlop et al. 2020, World Spider Catalog 2022). The genus *Plynnon* was established by Deeleman-Reinhold in 2001, based on the type species *P.jaegeri* Deeleman-Reinhold, 2001 and two other species. In Deeleman-Reinhold’s original diagnosis, she made a detailed distinction between *Plynnon* and its similar genus *Orthobula*, which was then in the subfamily Phrurolithinae of Corinnidae, but has now been transferred to Trachelidae.

Members of *Plynnon* are small to medium sized (2–5 mm), dark to reddish-brown spiders. *Plynnon* can be distinguished from other phrurolithid genera by the broad white band in the middle or distal half of tibia I, absence of spines on all the femora, absence of a tibial apophysis, conductor and median apophysis in the male palp, the special transverse copulatory ducts in the female vulva (Deeleman-Reinhold 2001), cheliceral anterior spines absent and the anterior metatarsi with only one pair of ventral spines (vs. at least two pairs of ventral spines in other phrurolithids).

Here, one new species of this genus is described from Yunnan Province, south-western China.

## Materials and methods

All measurements are given in millimetres (mm). Leg measurements are shown as: total length (femur, patella, tibia, metatarsus, tarsus). Total length is the sum of the carapace and abdomen lengths. Epigynes were removed and cleared in a pancreatin solution (Álvarez-Padilla and Hormiga 2007), transferred to alcohol and temporarily mounted for illustration. All specimens are preserved in 75% alcohol and were examined, illustrated and measured with a Leica M205A stereomicroscope, equipped with an Abbe drawing device. Photographs were taken using a Leica M205A stereomicroscope, equipped with a DFC550 CCD camera. The distribution map was downloaded from China Map Press (www.chinamap.com) and edited in CorelDRAW® Graphics Suite 12. The specimens used in this study are deposited in the Museum of Hebei University, Baoding, China (MHBU).

Abbreviations: **AER**, anterior eye row; **ALE**, anterior lateral eye; **AME**, anterior median eye; **B**, bursa; **CD**, copulatory duct; **CO**, copulatory opening; **CRW**, width of cephalic region at PLE; **CT**, connecting tube; **E**, embolus; **FDB**, femoral distal boss; **MOA**, median ocular area; **OAW**, width of ocular area; **PER**, posterior eye row; **PLE**, posterior lateral eye; **PME**, posterior median eye; **S**, spermatheca. Leg spination includes the following abbreviations: **plv**, prolateral ventral; **rlv**, retrolateral ventral.

## Taxon treatments

### Plynnon aduncum

Jin, Li & Zhang sp. n.

D4FDFE48-F802-564D-9624-85A1F6839135

32B7CC12-149A-484D-9689-FC7B9B104BEE

#### Materials

- **Type status:** Holotype. **Taxon:** scientificName: *Plynnonaduncum*; order: Araneae; family: Phrurolithidae; genus: Plynnon; **Location:** country: China; stateProvince: Yunnan; county: Tengchong; municipality: Baoshan; locality: Shangying Village; verbatimElevation: 1479; verbatimLatitude: 25.018151°N; verbatimLongitude: 98.687632°E; **Event:** year: 2017; month: 6; day: 10
- **Type status:** Paratype. **Taxon:** scientificName: *Plynnonaduncum*; order: Araneae; family: Phrurolithidae; genus: Plynnon; **Location:** country: China; stateProvince: Yunnan; county: Tengchong; municipality: Baoshan; locality: Shangying Village; verbatimElevation: 1479; verbatimLatitude: 25.018151°N; verbatimLongitude: 98.687632°E; **Event:** year: 2017; month: 6; day: 11

#### Description

**Figure 1.**
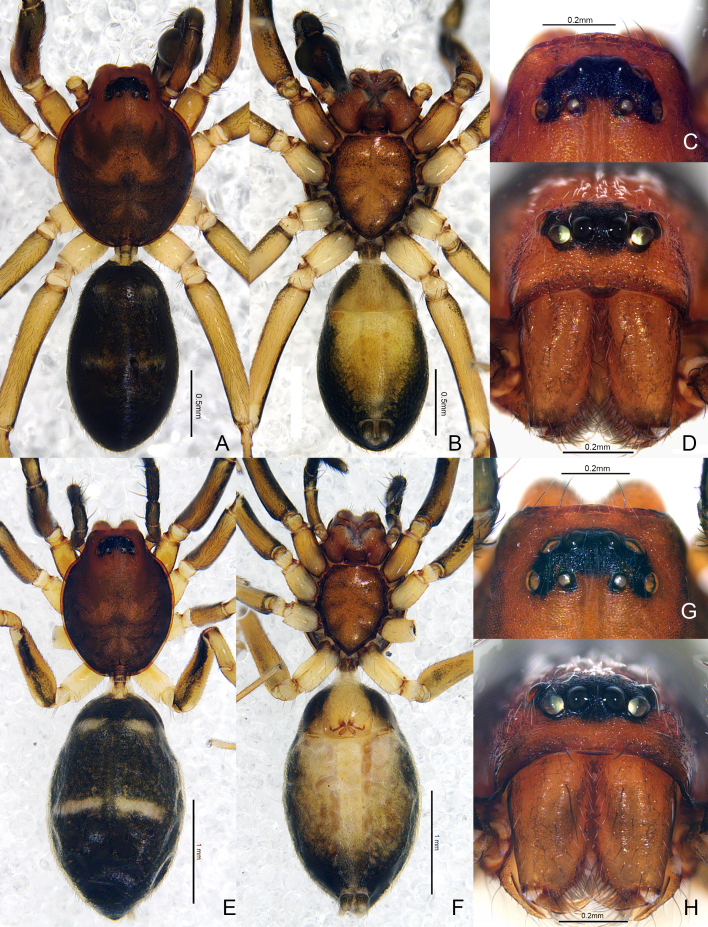
*Plynnonaduncum* sp. n. **A** male habitus, dorsal view; **B** same, ventral view; **C** male ocular area, dorsal view; **D** male cephalothorax, frontal view; **E** female habitus, dorsal view; **F** same, ventral view; **G** female ocular area, dorsal view; **H** female cephalothorax, frontal view.

**Figure 2.**
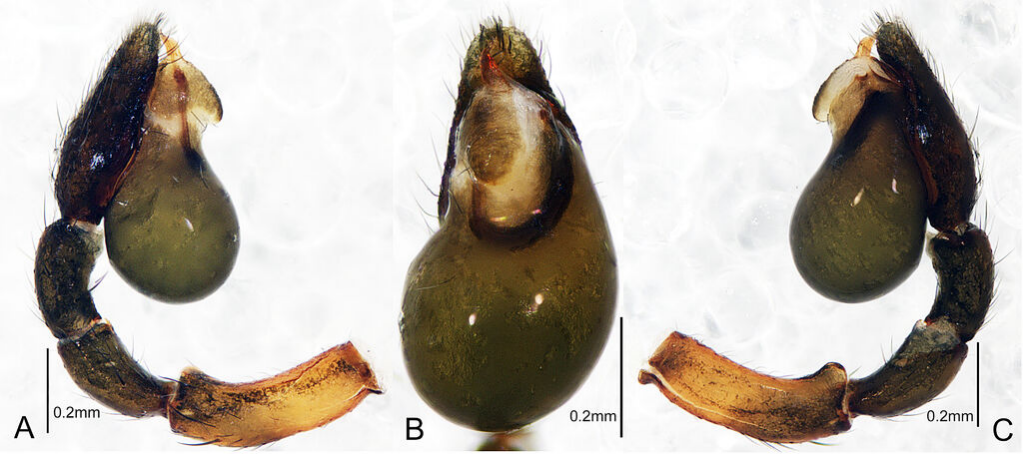
*Plynnonaduncum* sp. n. **A** male left palp, prolateral view; **B** same, ventral view; **C** same, retrorolateral view.

Male (holotype) (Fig. 1A–D, Fig. 2 and Fig. 4A–C). Total length 2.92: carapace 1.36 long, 1.07 wide, width/length 0.79; abdomen 1.56 long, 0.85 wide. Carapace oval, reddish-brown, with large dark markings on middle and rear; thoracic groove absent and fovea indistinct (Fig. 1A). Ocular area black; AER and PER recurved in dorsal view, AER slightly procurved in front view (Fig. 1C, D). Eye sizes and interdistances: AME 0.07, ALE 0.07, PME 0.04, PLE 0.07; AME–AME 0.04, AME–ALE 0.01, ALE–ALE 0.18, PME–PME 0.10, PME–PLE 0.05, PLE–PLE 0.28, ALE–PLE 0.05. OAW 0.37, CRW 0.53, OAW/CRW 0.70. CRW/carapace width 0.50. MOA 0.16 long, front width 0.17, back width 0.20. Clypeus height 0.14. Chilum (Fig. 1D) semicircular, sclerotized and dark brown. Chelicerae reddish-brown, anterior spines absent, with two promarginal and two retromarginal teeth. Endites brown. Labium dark brown and as wide as long (Fig. 1B). Sternum yellowish-brown, longer than wide, with dark margins and dark ribbed pattern in middle, precoxal triangles and intercoxal sclerites distinct (Fig. 1B). Leg I dark brown, with broad white band on 2/3 of tibia distally; legs II–IV light yellow, with balck stripes present on both sides of femora, patellae, tibiae and metatarsi. Measurements of legs: leg I 3.23 (0.93 + 0.37 + 0.77 + 0.64 + 0.52), II 3.01 (0.84 + 0.35 + 0.65 + 0.63 + 0.54), III 2.81 (0.77 + 0.34 + 0.58 + 0.61 + 0.51), IV 4.21 (1.14 + 0.14 + 0.92 + 1.00 + 0.74). Leg formula: 4123. Leg spination: tibiae: I plv 4 rlv 4, II plv 4 rlv 3; metatarsi: I–II plv 1 rlv 1. Abdomen long oval, black; anterior 2/3 with a narrow dorsal scutum and two pairs of inconspicuous transverse white bands (Fig. 1A).

Palp as illustrated (Fig. 2 and Fig. 4A–C). Femur with a cup-shaped retrolateral distal boss; patellar and tibial apophyses absent. Tegulum pyriform, with an ovate subapical protrusion; sperm duct U-shaped and short, only present in apical half of tegulum. Embolus beak-shaped, with sharp tip pointed retrolaterally. Cymbium apically with a tuft of thick, flat setae around the apex of embolus.

**Figure 3.**
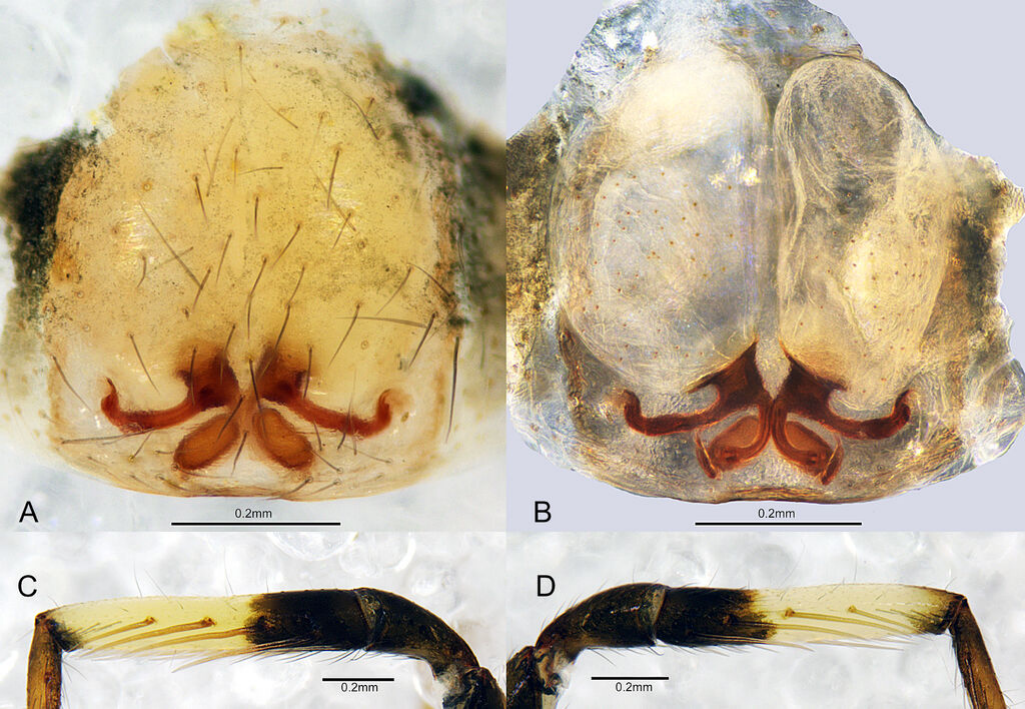
*Plynnonaduncum* sp. n. **A** epigyne, ventral view; **B** vulva, dorsal view; **C** female tibia I, prolateral view; D same, retrolateral view.

**Figure 4.**
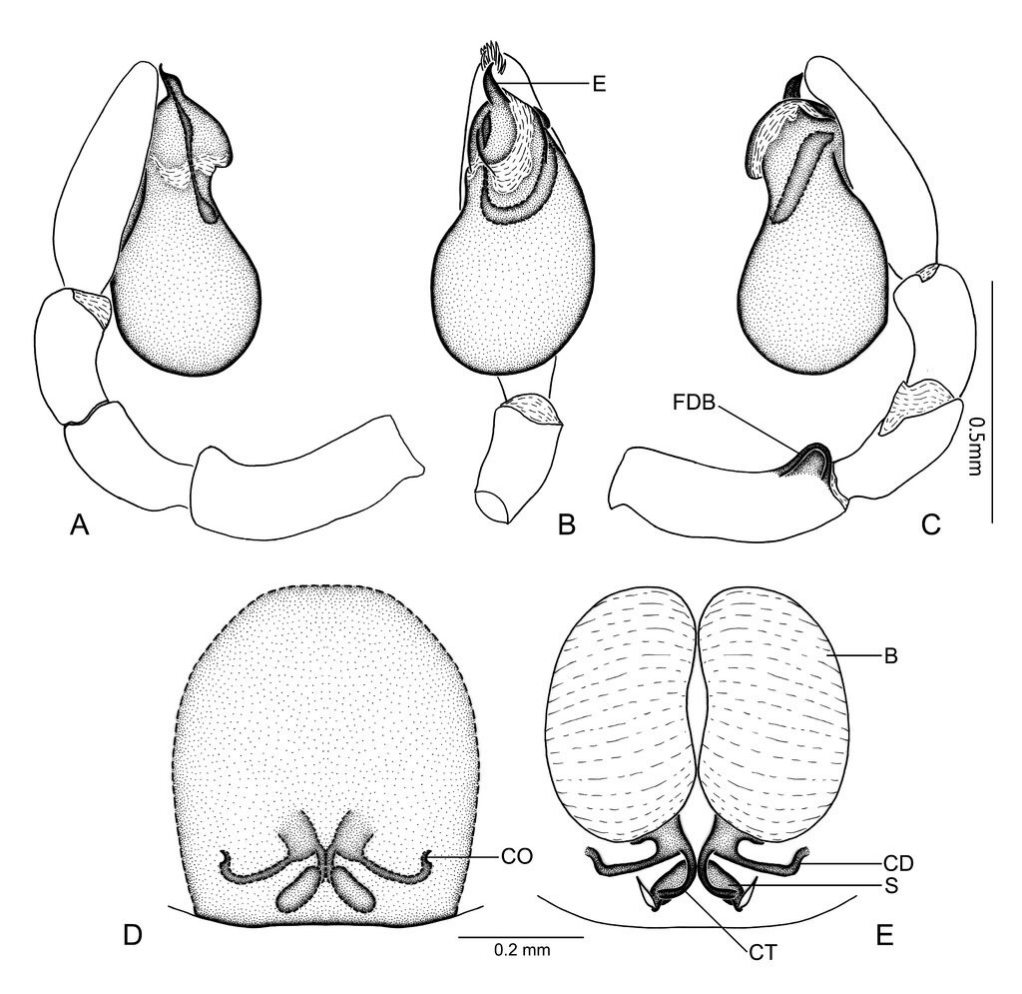
*Plynnonaduncum* sp. n. **A** male left palp, prolateral view; **B** same, ventral view; **C** same, retrorolateral view; **D** epigyne, ventral view; **E** vulva, dorsal view. Abbreviations: B, bursa; CD, copulatory duct; CO, copulatory opening; CT, connecting tube; E, embolus; FDB, femoral distal boss; S, spermatheca.

Female (Fig. 1E–H, Fig. 3 and Fig. 4D–E). Total length 3.61: carapace 1.42 long, 1.10 wide, width:length = 0.77; abdomen 2.19 long, 1.35 wide. Eye sizes and interdistances: AME 0.06, ALE 0.07, PME 0.04, PLE 0.07; AME–AME 0.05, AME–ALE 0.01, ALE–ALE 0.19, PME–PME 0.13, PME–PLE 0.05, PLE–PLE 0.31, ALE–PLE 0.06. OAW 0.39, CRW 0.69, OAW: CRW= 0.57. CRW: carapace width = 0.62. MOA 0.17 long, front width 0.17, back width 0.21. Clypeus height 0.12, 2 times AME diameter. Leg I dark brown, with broad white band on two-thirds of tibia distally (Fig. 3C–D). Measurements of legs: leg I 3.69 (1.06 + 0.42 + 0.89 + 0.69 + 0.63), II 3.38 (0.98 + 0.37 + 0.76 + 0.66 + 0.61), III 3.11 (0.85 + 0.37 + 0.66 + 0.68 + 0.55), IV 3.83 (1.33 + 0.42 + 0.11 + 1.17 + 0.80). Leg formula: 4123. Leg spination: tibiae: I plv 5 rlv 4, II plv 4 rlv 3; metatarsi: I–II plv 1 rlv 1. Abdomen with conspicuous transverse white bands (Fig. 1E). Other somatic characters as in male.

Epigyne (Fig. 3A and Fig. 4D): poorly sclerotized, spermathecae and copulatory ducts visible through translucent cuticle; copulatory openings small, located posteriorly, separated by about four spermatheca lengths. Vulva (Fig. 3B and Fig. 4E): copulatory ducts directed laterally, anterior part hook-shaped; bursae large, kidney-shaped, with sclerotized bases; spermathecae long oval, close to each other; connecting tubes curved, connecting bursal bases and spermathecae.

#### Diagnosis

The new species is similar to *Plynnonzborowskii* in having a pyriform palpal tegulum and the long oval spermathecae that have similar position, but can be distinguished by: 1) embolus beak-shaped (Fig. 2B and Fig. 4B) vs. S-shaped in *P.zborowskii* (Deeleman-Reinhold 2001: 440, fig. 718); 2) femoral distal boss of male palp present (Fig. 2C and Fig. 4C) vs. absent in *P.zborowskii* (Deeleman-Reinhold 2001: 440, fig. 719); 3) the position of copulatory openings is almost parallel to the bases of the bursae (Fig. 3A and Fig. 4D) vs. noticeably posterior in *P.zborowskii* (Deeleman-Reinhold 2001: 440, fig. 720); 4) the middle part of the copulatory ducts almost straight (Fig. 3B and Fig. 4E) vs. folded in *P.zborowskii* (Deeleman-Reinhold 2001: 440, fig. 720).

#### Etymology

The specific name is a Latin adjective meaning “hooked”. It refers to the front part of copulatory ducts curved as hook-shaped.

#### Distribution

**Figure 5.**
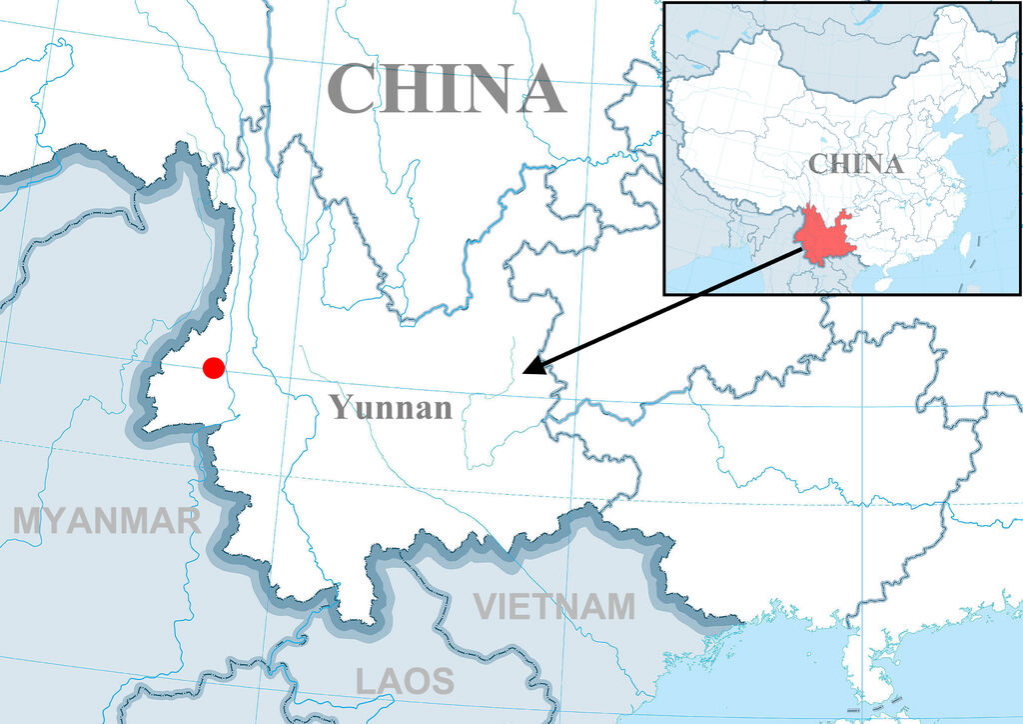
Distribution of *Plynnonaduncum* sp. n. (red circle).

Known only from the type locality (Yunnan, China) (Fig. 5).

## Discussion

In our opinion, the genus *Plynnon* is correctly assigned to Phrurolithidae rather than Trachelidae, considering the spination pattern in both sexes: the presence of pairs of strong ventral spines on the anterior tibiae and metatarsi and combination of the absence of any ventral spines or cusps on the anterior tarsi. By contrast, in Trachelidae, there are three conditions: 1) the anterior legs without any spines and cusps, such as *Trachelasminor* O. Pickard-Cambridge, 1872, the type species of the genus *Trachelas* L. Koch, 1866 (Jin et al. 2017); 2) the anterior tibiae, metatarsi and tarsi all with pairs of strong ventral spines, such the genus *Orthobula* Simon, 1897 (Deeleman-Reinhold 2001); 3) the anterior tarsi with ventral cusps, at the same time, the anterior metatarsi and tibiae with cusps or spines, such as the genus *Spinotrachelas* Haddad, 2006 (Haddad 2006) and *Cetonana* Strand, 1929 (Jin et al. 2017b).

## Supplementary Material

XML Treatment for Plynnon aduncum
